# Supplementary material for: CTRP3 attenuates inflammation, oxidative and cell death in cisplatin induced HK-2 cells
Source: PeerJ. 2023 Aug 23;11:e15890. doi: 10.7717/peerj.15890 (PMC10460153; doi:10.7717/peerj.15890)
Supplement: Supplemental Information 3 [file peerj-11-15890-s003.pptx]

## Slide 1
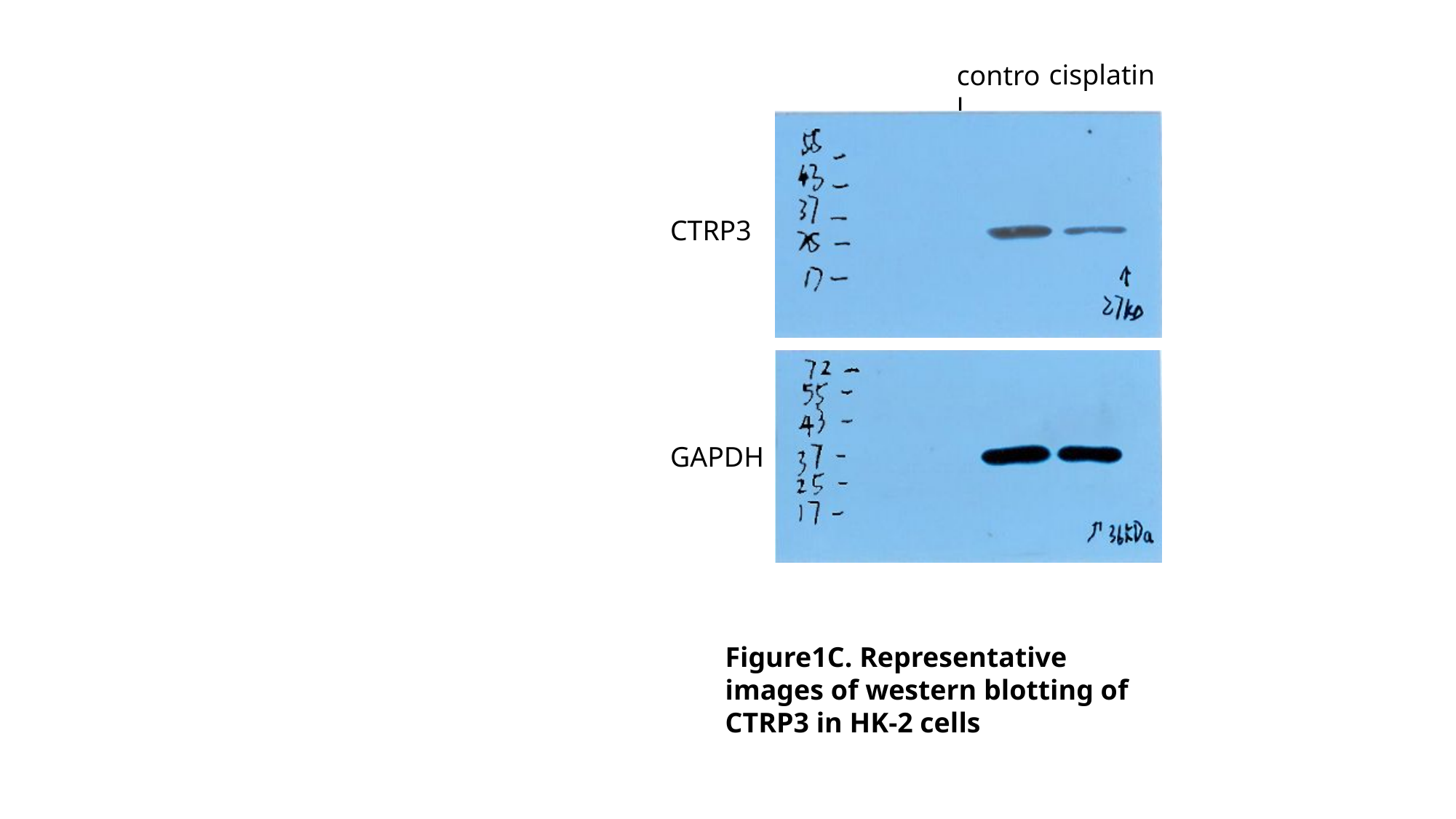

cisplatin
control
CTRP3
GAPDH
Figure1C. Representative images of western blotting of CTRP3 in HK-2 cells

## Slide 2
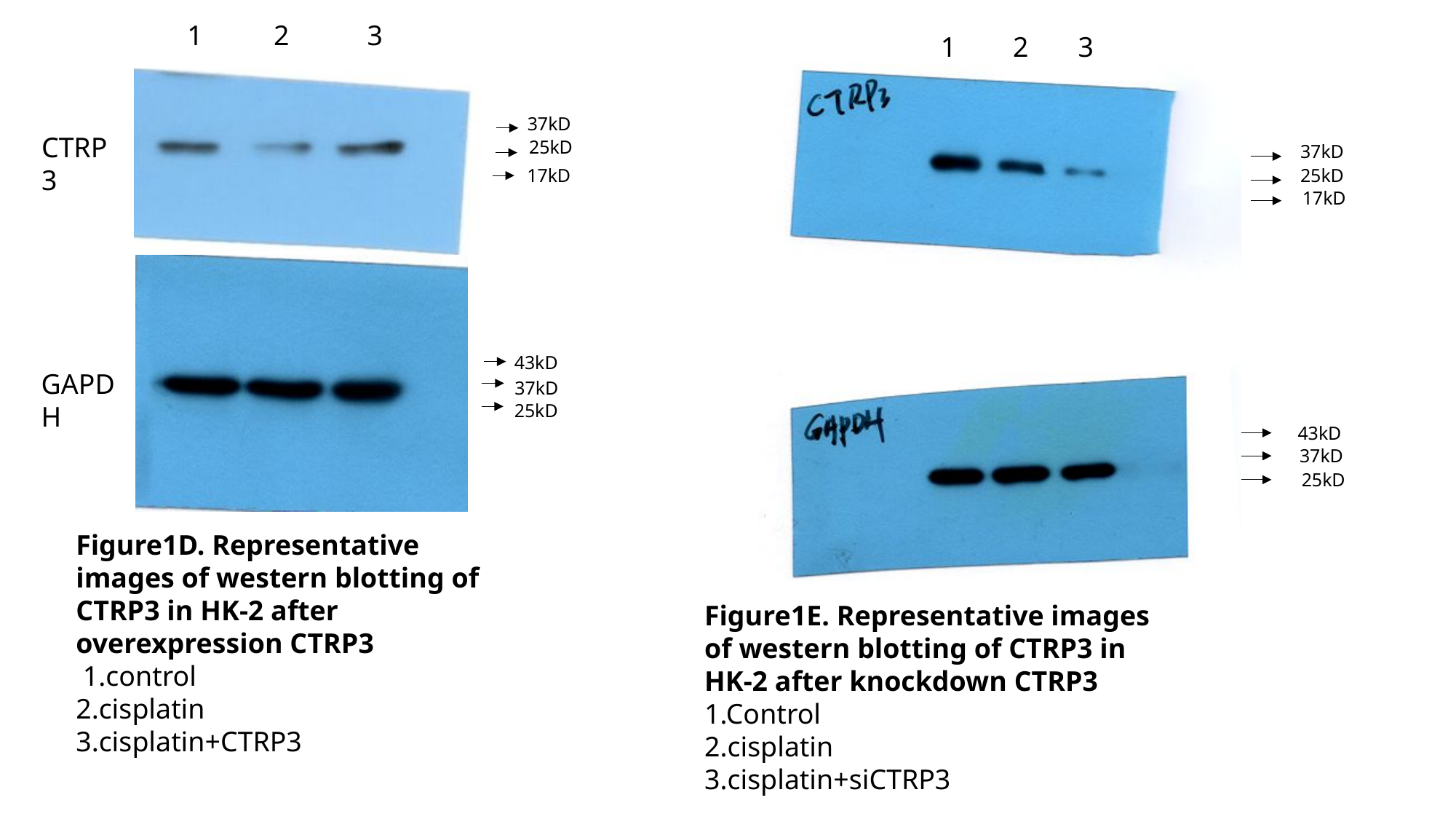

1 2 3
 1 2 3
37kD
CTRP3
25kD
37kD
17kD
25kD
17kD
43kD
GAPDH
37kD
25kD
43kD
37kD
25kD
Figure1D. Representative images of western blotting of CTRP3 in HK-2 after overexpression CTRP3
 1.control
2.cisplatin
3.cisplatin+CTRP3
Figure1E. Representative images of western blotting of CTRP3 in HK-2 after knockdown CTRP3
1.Control
2.cisplatin
3.cisplatin+siCTRP3

## Slide 3
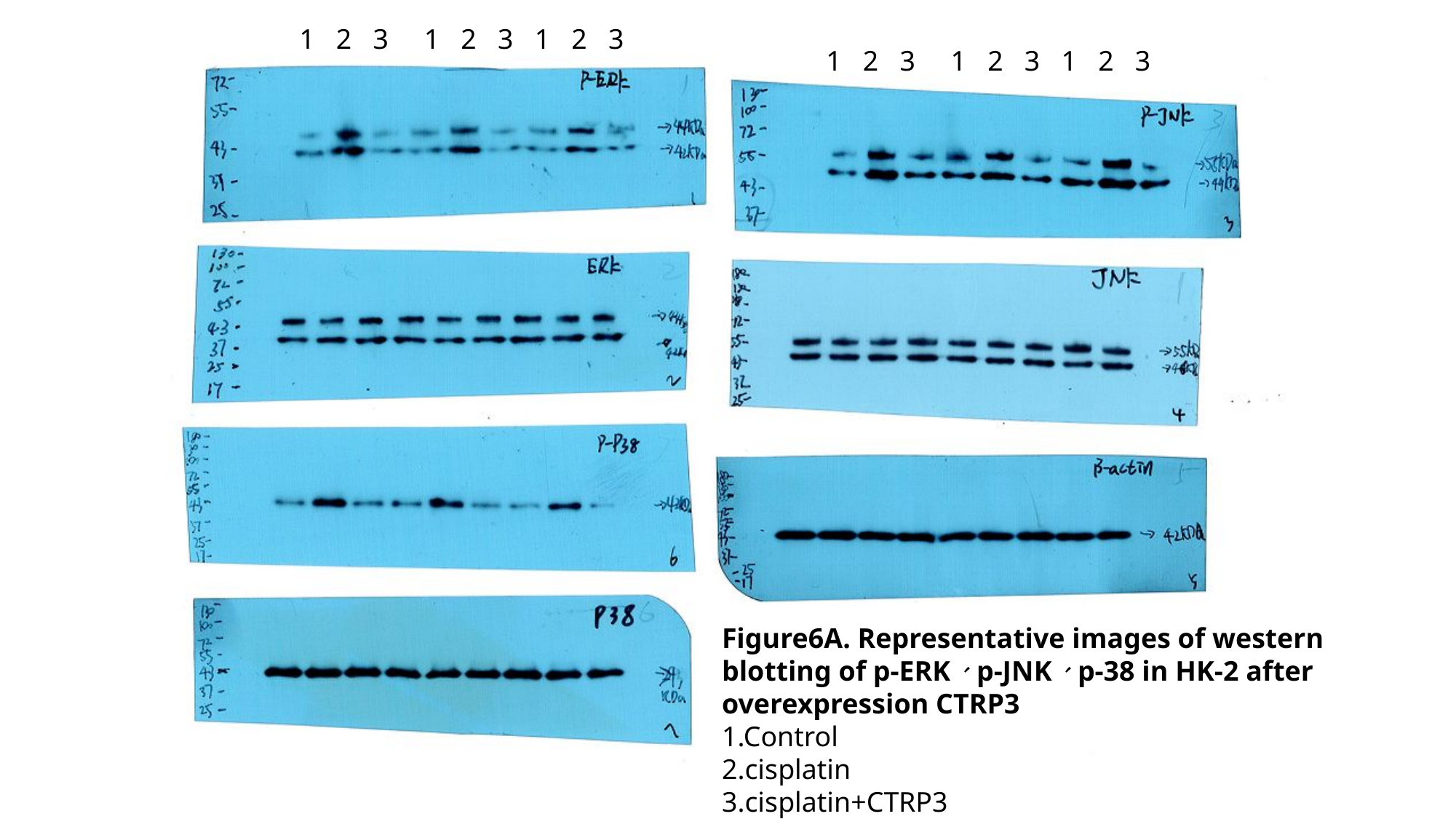

1 2 3 1 2 3 1 2 3
1 2 3 1 2 3 1 2 3
Figure6A. Representative images of western blotting of p-ERK、p-JNK、p-38 in HK-2 after overexpression CTRP3
1.Control
2.cisplatin
3.cisplatin+CTRP3

## Slide 4
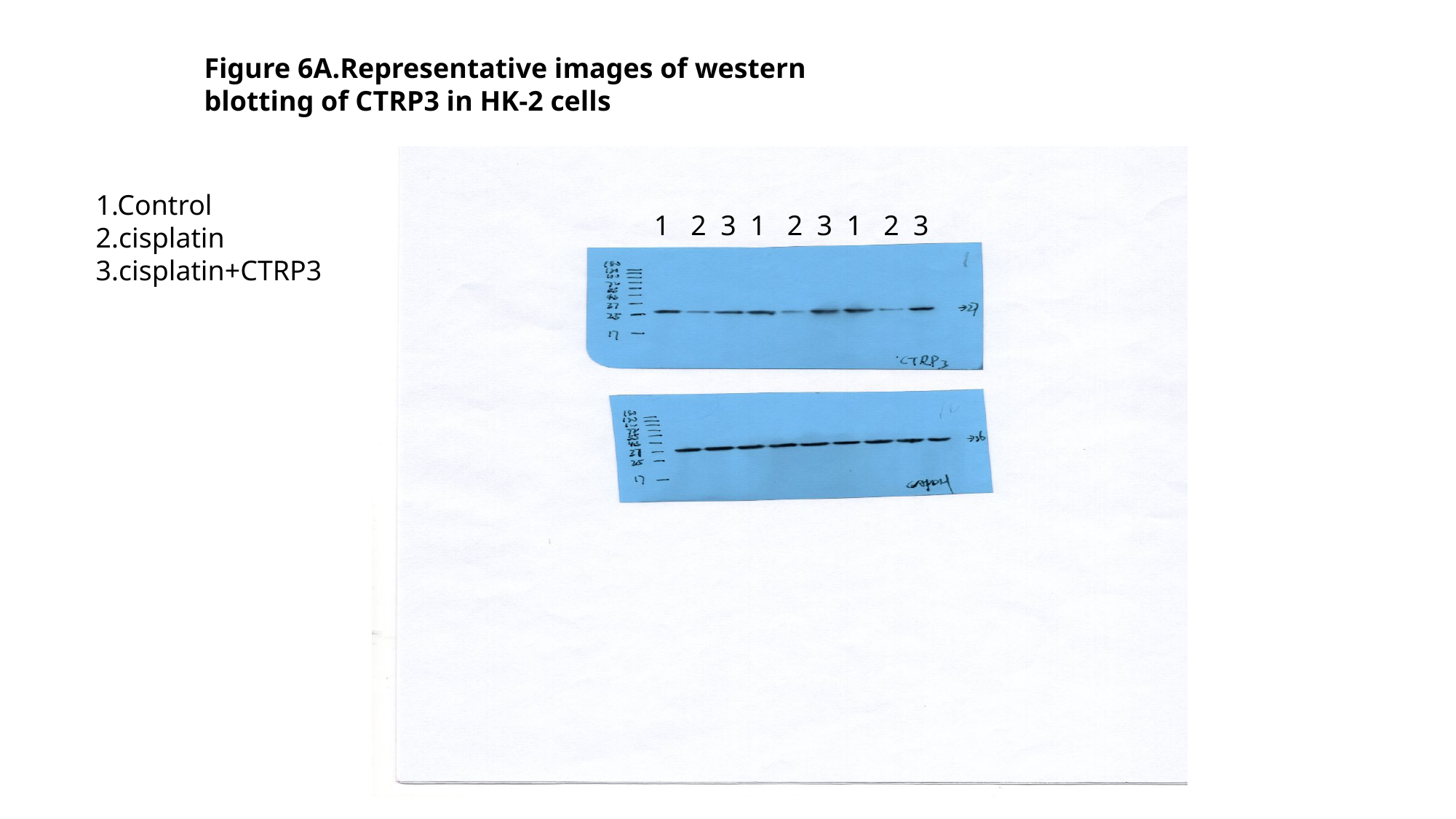

Figure 6A.Representative images of western blotting of CTRP3 in HK-2 cells
#
1.Control
2.cisplatin
3.cisplatin+CTRP3
1 2 3 1 2 3 1 2 3

## Slide 5
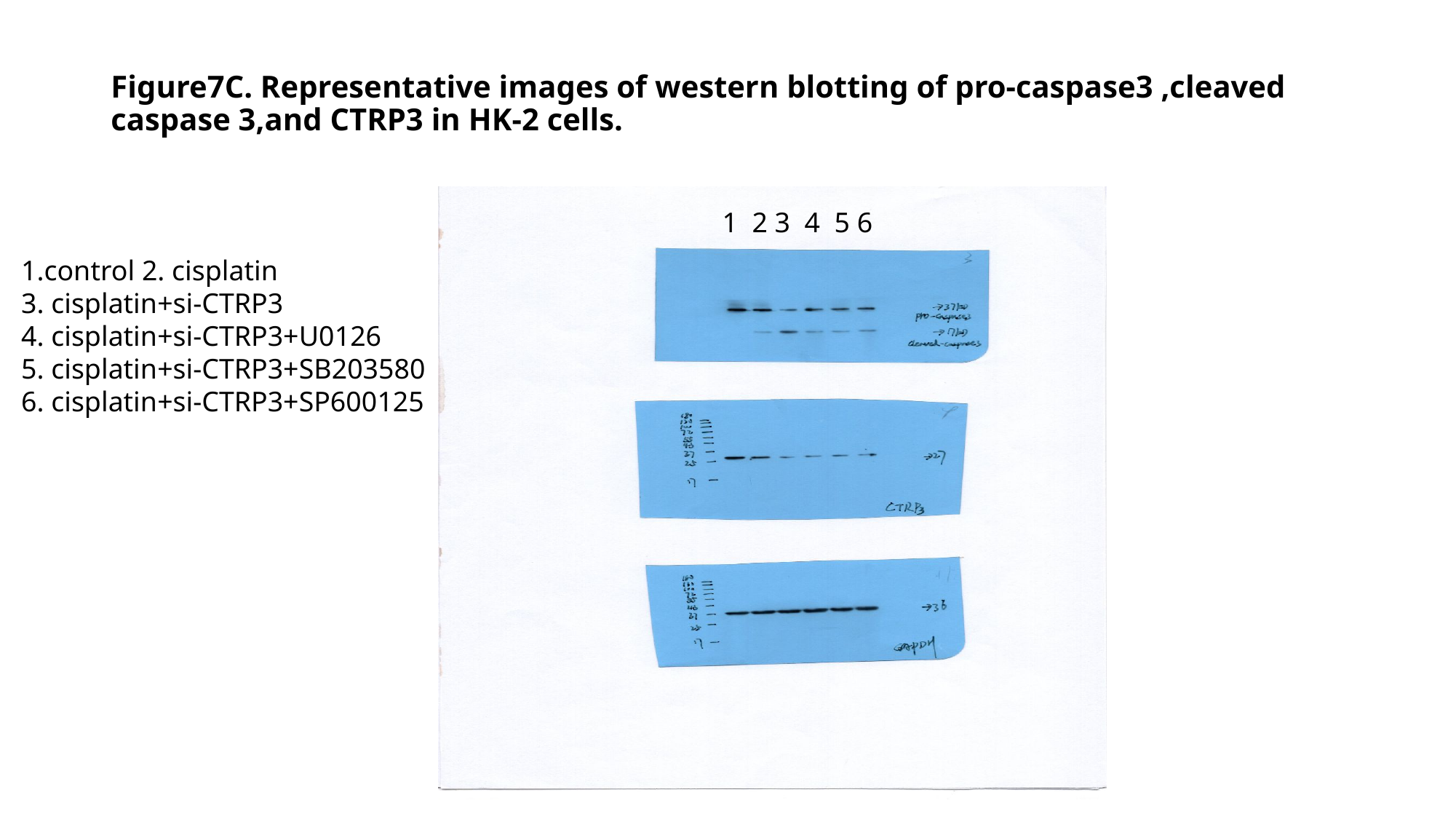

# Figure7C. Representative images of western blotting of pro-caspase3 ,cleaved caspase 3,and CTRP3 in HK-2 cells.
1 2 3 4 5 6
1.control 2. cisplatin
3. cisplatin+si-CTRP3
4. cisplatin+si-CTRP3+U0126
5. cisplatin+si-CTRP3+SB203580
6. cisplatin+si-CTRP3+SP600125

## Slide 6
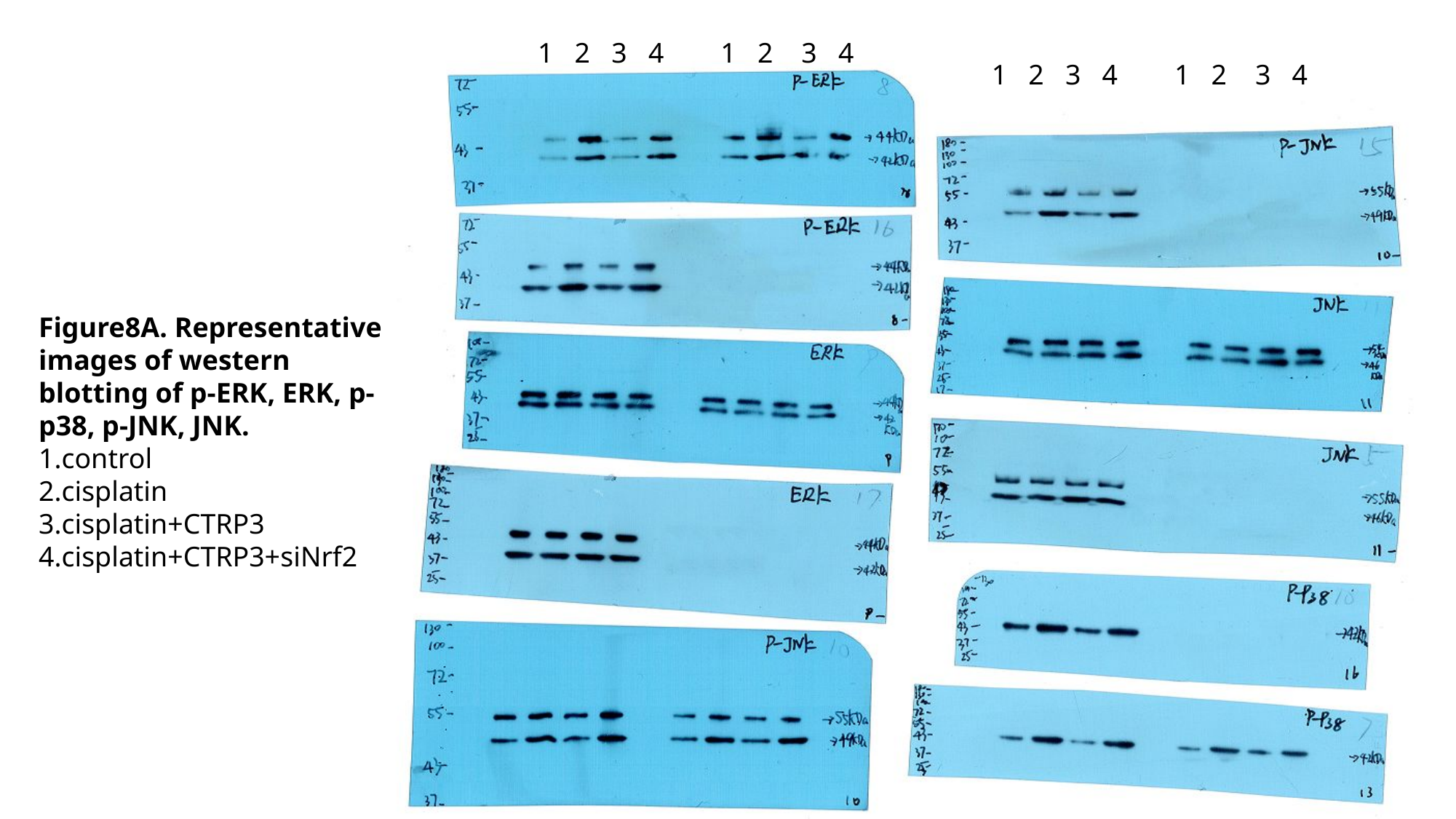

1 2 3 4 1 2 3 4
 1 2 3 4 1 2 3 4
Figure8A. Representative images of western blotting of p-ERK, ERK, p-p38, p-JNK, JNK.
1.control
2.cisplatin
3.cisplatin+CTRP3
4.cisplatin+CTRP3+siNrf2

## Slide 7
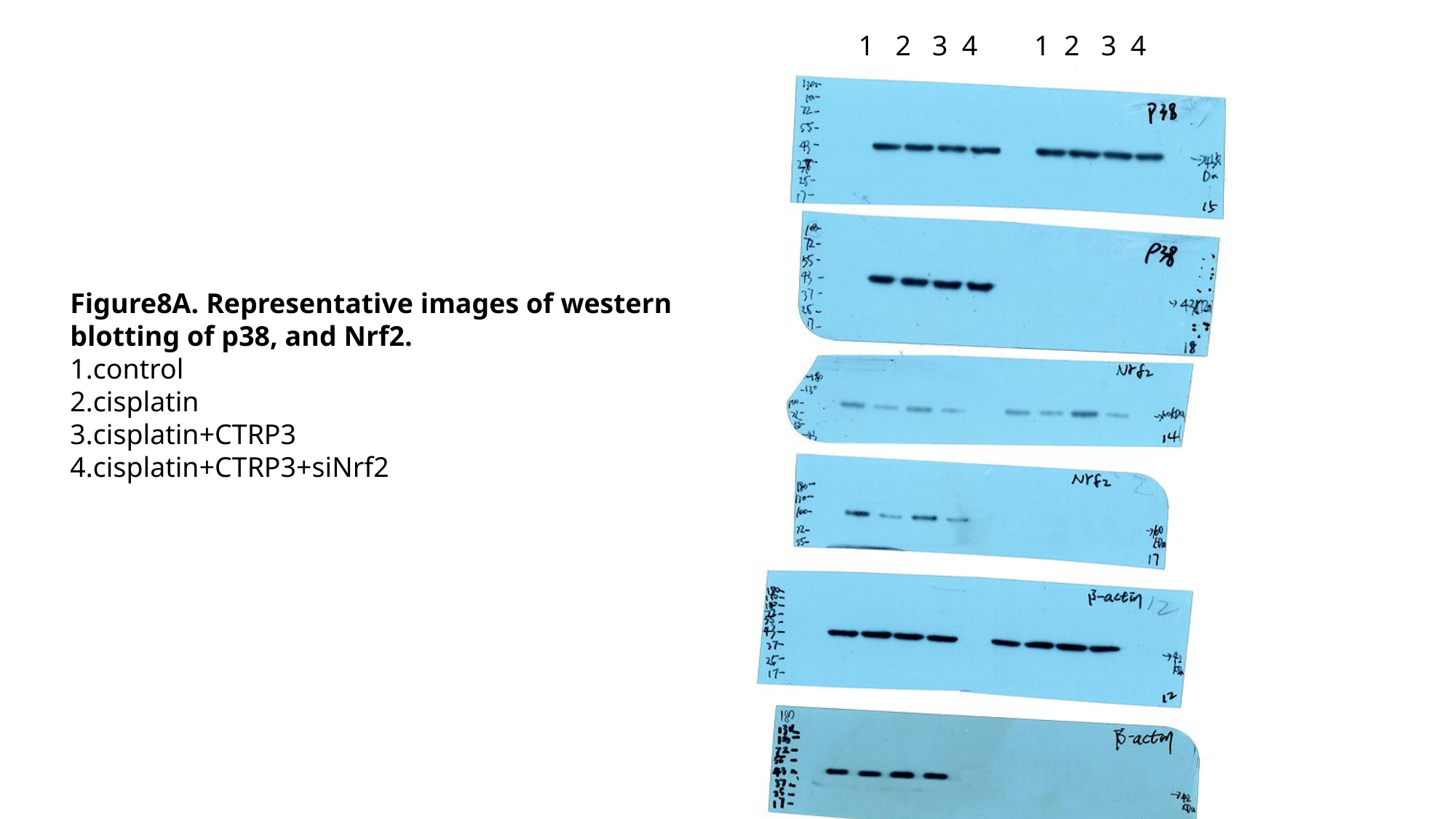

1 2 3 4 1 2 3 4
Figure8A. Representative images of western blotting of p38, and Nrf2.
1.control
2.cisplatin
3.cisplatin+CTRP3
4.cisplatin+CTRP3+siNrf2

## Slide 8
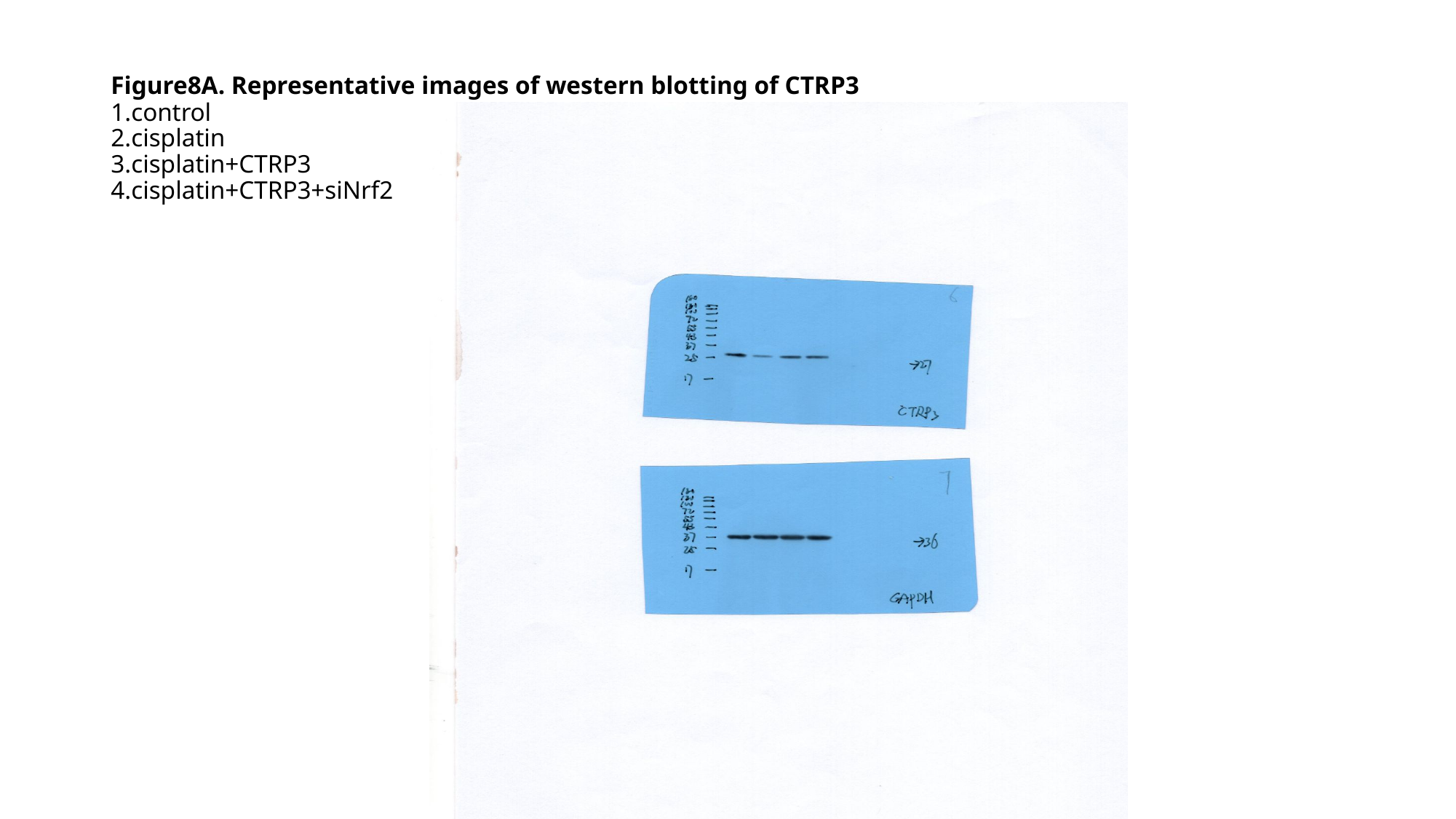

# Figure8A. Representative images of western blotting of CTRP3 1.control 2.cisplatin 3.cisplatin+CTRP3 4.cisplatin+CTRP3+siNrf2
